# Neuroprotective potential of the natural polyphenol Procyanidin B2 in spinal cord injury: a comprehensive study utilizing machine learning, network pharmacology, and *in vivo* validation

**DOI:** 10.3389/fnut.2026.1872188

**Published:** 2026-07-06

**Authors:** Chunyu Xiang, Yang Liu, Rui Gu, Wanguo Liu, Jingwei Shi

**Affiliations:** 1 Department of Laboratory Medicine Center, China-Japan Union Hospital of Jilin University, Changchun, China; 2 Department of Orthopedic Surgery, China-Japan Union Hospital of Jilin University, Changchun, China

**Keywords:** bioinformatics, caspase-1, inflammation, natural polyphenols, neuroprotection, Procyanidin B2, spinal cord injury

## Abstract

**Background:**

The secondary injury cascade following spinal cord injury (SCI) drives severe inflammation and tissue destruction. Although the natural polyphenol Procyanidin B2 (PCB2) has well-documented neuroprotective properties, its specific therapeutic efficacy in SCI, as well as its precise therapeutic targets and immunomodulatory mechanisms, remain unclear.

**Methods:**

We applied an integrated *bioinformatics* and *in vivo* approach. Target predictions were cross-referenced with SCI transcriptomic profiles from GEO datasets. Four machine learning algorithms were used to isolate core regulatory genes. Single-cell RNA sequencing mapped the primary target's distribution, and molecular docking estimated binding affinities. Mechanistic predictions were validated in a rat T10 spinal cord contusion model via Basso–Beattie–Bresnahan (BBB) scoring, histology, immunofluorescence, and Western blot.

**Results:**

Network pharmacology initially yielded 59 shared targets, with functional enrichment pointing to PCB2's broad involvement in Toll-like receptor and p53 signaling, as well as tissue remodeling. This suggests its potential for multi-target anti-inflammatory and anti-apoptotic intervention. Machine learning algorithms then pinpointed Caspase-1 (CASP1) as the central regulatory node. Single-cell RNA sequencing showed that CASP1 expression surges specifically within macrophages and microglia following injury. Molecular docking supported a robust interaction (−7.2 kcal/mol) between PCB2 and the active pocket of CASP1. In our rat model, administering PCB2 notably hastened the return of bladder control and increased BBB locomotor scores. Histology confirmed that treated animals had smaller lesion volumes, better myelin integrity, and less inflammatory cell infiltration. At the molecular level, PCB2 significantly suppresses CASP1 expression, thereby blunting secondary damage.

**Conclusion:**

PCB2 demonstrates significant neuroprotective effects in SCI. Its mechanism primarily involves targeting *CASP1* in myeloid cells, to lower its expression, thereby shifting the microenvironment toward repair, providing a translational basis for utilizing natural polyphenols like PCB2 in managing secondary spinal cord trauma and supporting overall central nervous system health.

## 1 Introduction

Spinal cord injury (SCI) causes permanent motor, sensory, and autonomic deficits, creating a significant global socioeconomic burden (1, 2). After the initial mechanical trauma, the lesion site experiences a destructive secondary injury cascade. Severe inflammation, oxidative stress, apoptosis, and pyroptosis dominate this microenvironment, progressively worsening neuronal loss and impairing tissue repair (3, 4). Clinical management currently relies on surgical decompression and symptomatic support (5). High-dose methylprednisolone, a common pharmacological intervention, remains controversial because of its limited efficacy and severe systemic side effects (6). Since secondary SCI involves intertwined pathological networks rather than a single targetable pathway, conventional single-target drugs often fail to yield satisfactory outcomes. Clinicians urgently need effective, low-toxicity, multi-target neuroprotective agents to suppress these secondary cascades and support functional recovery.

Researchers increasingly view natural compounds as strong candidates for multi-target drug development, largely because of their structural diversity, broad biological activity, and favorable safety profiles (7–10). Procyanidin B2 (PCB2), a natural polyphenol and flavonoid dimer formed by two epicatechin units with a C4–C8 bond, occurs naturally in grape seeds, apple peels, and cocoa. Accumulating evidence highlights the potential of such polyphenols in promoting central nervous system health and neuroprotection. Multiple phenolic hydroxyl groups in its structure provide strong free radical scavenging and antioxidant capacity (11). Preclinical studies show that PCB2 offers neuroprotection in several central nervous system disorders. In models of multiple sclerosis, Alzheimer's disease, and cerebral ischemia, PCB2 reduces neurological damage and helps preserve motor and cognitive functions. It achieves this by neutralizing reactive oxygen species (ROS), reducing inflammatory cytokine release, and modulating apoptosis (12–14). These anti-inflammatory and tissue-preserving properties, which are also observed in other natural polyphenols (15), suggest PCB2 could counter the complex secondary injury cascades of SCI. However, its specific therapeutic efficacy in SCI is not well documented, and researchers have yet to systematically investigate the precise molecular targets and network regulatory mechanisms it affects within the injured spinal cord microenvironment.

Researchers frequently use network pharmacology to explore the mechanisms of natural compounds. Traditional computational predictions, however, depend heavily on static databases and blind docking. These approaches often produce false-positive interactions and lack disease-specific biological context (16), limiting their translation into clinical practice. Integrating multi-omics data with machine learning algorithms offers a more precise strategy for target discovery (17). Caspase-1 (*CASP1*) and inflammasome-mediated immune responses contribute to tissue deterioration during the secondary phase of SCI (18–20). Identifying *CASP1* as the core functional target of PCB2 in this heterogeneous microenvironment demands robust methodology. We can eliminate false-positive noise and map the core therapeutic targets of PCB2 to specific cellular populations—such as infiltrating immune cells—by combining multi-algorithm machine learning for dimensionality reduction with single-cell RNA sequencing (scRNA-seq) for spatial localization.

In this study, we established a closed-loop research paradigm to systematically evaluate the therapeutic efficacy and molecular mechanisms of PCB2 in SCI. Our workflow integrated multi-database target prediction with multi-algorithm machine learning to screen and identify *CASP1* as the core regulatory target. We then coupled this approach with scRNA-seq to determine the spatial localization of *CASP1* within the immune microenvironment, and supported these findings with molecular docking simulations. Finally, we conducted *in vivo* validation using a rat SCI contusion model to confirm the phenotypic outcomes. By verifying the *bioinformatics* analyses with comprehensive wet-lab data, we show that PCB2 promotes functional recovery and tissue repair by suppressing the *CASP1*-driven secondary injury cascade. These findings clarify the neuroprotective network of PCB2 and offer a translational basis for its clinical application in spinal cord trauma.

## 2 Materials and methods

### 2.1 Overall research framework

**Figure 1:**
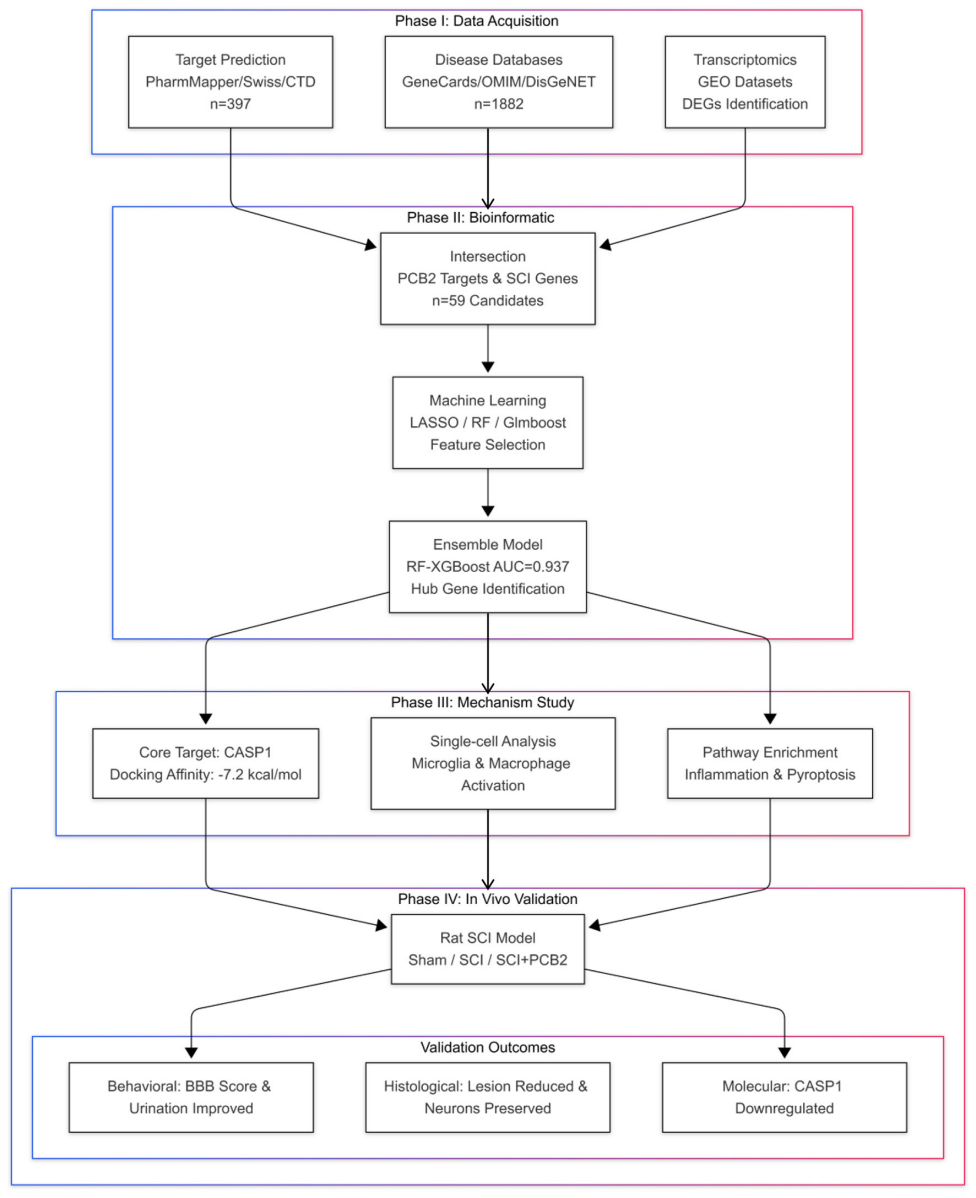
Flowchart of the research.

The research workflow, depicted in Figure 1, integrates bioinformatics screening with experimental validation. Multiple databases were initially mined to predict potential pharmacological targets for PCB2. In parallel, SCI-related genes were extracted from established disease databases and relevant transcriptome datasets. By overlapping these gene sets and applying machine learning algorithms, the core candidate targets were isolated. Finally, a rat SCI model alongside molecular biology assays was employed to verify whether PCB2 regulates *CASP1 in vivo*.

### 2.2 Data source and acquisition

Transcriptomic data related to SCI (GSE5296, GSE47681, GSE42828, GSE45006, and GSE189070) were downloaded from the Gene Expression Omnibus (GEO) (https://www.ncbi.nlm.nih.gov/geo/). To test the generalization of our machine learning models, an independent in-house dataset comprising 12 rat spinal cord samples (6 SCI and 6 Sham) from prior work was incorporated. For target prediction, PharmMapper (http://www.lilab-ecust.cn/pharmmapper/), SwissTargetPrediction (http://www.swisstargetprediction.ch/), and the Comparative Toxicogenomics Database (CTD, https://ctdbase.org/) were searched to identify putative binding targets of PCB2. Additionally, GeneCards (https://www.genecards.org/), OMIM (https://omim.org/), and DisGeNET (https://www.disgenet.org/) were queried using “spinal cord injury” to assemble a comprehensive list of disease-associated genes.

### 2.3 Experimental animals and reagents

All animal protocols received approval from the Animal Ethics Committee of Jilin University. Specific pathogen-free (SPF) female Sprague-Dawley (SD) rats (8–10 weeks old, 200–250 g) were sourced from the universitys Experimental Animal Center. The animals were kept under standard laboratory conditions (23 ± 2°C, 50 ± 5% humidity, 12-h light/dark cycle) with *ad libitum* access to food and water, and were allowed to acclimate for 1 week before surgery. The study complies with ARRIVE 2.0 guidelines. Rats presenting with severe postoperative complications or incomplete initial paralysis were excluded. PCB2 (analytical standard, purity 98%, cat. no. 42157) was obtained from Sigma-Aldrich (St. Louis, MO, USA). For immunofluorescence, a primary antibody targeting *CASP1* (cat. no. ab179515) was purchased from Abcam (Cambridge, UK). For Western blotting, an antibody specifically recognizing the cleaved CASP1 p10 subunit (1:1,000, cat. no. AF4022) was obtained from Affinity Biosciences (Cincinnati, OH, USA). An anti-GAPDH antibody (1:5,000, cat. no. 5174S) from Cell Signaling Technology (Danvers, MA, USA) was used as a loading control. Secondary antibodies, specifically HRP-conjugated goat anti-rabbit IgG (1:5,000, cat. no. ab6721) and Alexa Fluor 594-conjugated goat anti-rabbit IgG (1:500, cat. no. ab150080), were supplied by Abcam.

### 2.4 Data preprocessing

Raw microarray data (from GSE5296, GSE47681, GSE42828, and GSE45006) underwent normalization via the robust multi-array average (RMA) method using the “affy” R package (v1.76.0) (21). Probes without mapped gene symbols were discarded, and expression values from multiple probes targeting the same gene were averaged. For the scRNA-seq dataset (GSE189070), quality control relied on the “Seurat” R package (v5.2.1) (22). Cells displaying over 15% mitochondrial gene content or extreme gene counts (< 200 or >7,500) were excluded (filtered by nFeature 200–7,500, nCount < 40,000, mt ≤ 15%, and integrated via Harmony). Canonical correlation analysis (CCA) was then implemented to mitigate batch effects and align the data.

### 2.5 Prediction of PCB2 target genes and screening of SCI-related genes

The Simplified Molecular Input Line Entry System (SMILES) string for PCB2, acquired from PubChem, was queried against PharmMapper and SwissTargetPrediction to predict direct binding targets (23). In addition, genes associated with “Procyanidin B2” were extracted from the CTD. These three target sets were merged in R, and duplicates were removed to yield a final list of unique PCB2 targets.

Simultaneously, SCI-related genes were compiled from GeneCards (filtering for relevance score ≥ 1), OMIM, CTD (inference score ≥ 20), and DisGeNET database. After deduplication in R, this comprehensive SCI gene set was mapped against the predicted PCB2 targets. The overlapping genes were designated as candidate targets for PCB2 intervention in SCI.

### 2.6 Differential expression gene (DEG) analysis

To minimize non-biological variance, the GSE5296 and GSE47681 datasets were merged and batch correction was performed (batch correction via ComBat). Principal component analysis (PCA), executed via the “factoextra” R package (v1.0.7), confirmed the successful elimination of batch effects. DEGs between the SCI and sham groups were subsequently identified using the “limma” R package (v3.54.0) (24). The selection thresholds were set at an absolute log2 fold change (|log2 FC|) ≥ 0.5 and an adjusted *P*-value (Padj) ≤ 0.05. Visualizations, including volcano plots, were generated using the “ggplot2” package (v3.5.1) (25).

### 2.7 Functional enrichment and protein-protein interaction (PPI) network analysis

The biological significance of the candidate genes was explored through Gene Ontology (GO) and Kyoto Encyclopedia of Genes and Genomes (KEGG) pathway enrichment analyses, utilizing the “clusterProfiler” R package (v4.6.2) with a significance cutoff of Padj ≤ 0.05 (26). A protein-protein interaction (PPI) network was constructed via the STRING database (v12.0, https://string-db.org/) by applying a minimum interaction score of 0.7. The resulting network was visualized and analyzed in Cytoscape (v3.10.2) (27).

### 2.8 Core target screening via machine learning and model validation

To pinpoint the most crucial targets, four distinct machine learning algorithms were applied: LASSO regression, Glmboost, stepwise regression, and random forest (28–31). The genes selected by each method were subsequently used to train predictive models distinguishing SCI from control samples. For model development, the dataset was randomly split into a 70% training cohort and a 30% validation cohort. Model performance was evaluated based on the area under the receiver operating characteristic curve (AUC). To interpret the weight of individual targets within the final model, SHapley Additive exPlanations (SHAP) values were computed using the “shapviz” R package (v0.9.2).

### 2.9 Molecular docking of PCB2 and CASP1

The 3D crystal structure of *CASP1* was retrieved from the Protein Data Bank (PDB, https://www.rcsb.org/), and PyMOL (v2.6.0) was used to strip away water molecules and native ligands. Concurrently, the 3D structure of PCB2 was downloaded from PubChem and prepared in ChemDraw (v21.0) through hydrogenation and charge assignment. Molecular docking was executed with AutoDock Vina (v1.1.2), specifically targeting the active site pocket of *CASP1* (32). The binding affinity was quantified by the docking score, with values ≤ −7.0 kcal/mol considered indicative of stable interaction. The resulting binding conformations were visualized in PyMOL.

### 2.10 CASP1-anchored co-expression network and immunological profiling

Using the GeneCOCOA framework, the functional network surrounding *CASP1* was delineated via comparative co-expression analysis (33). By modeling the correlation between *CASP1* expression and MSigDB Hallmark gene sets in the SCI cohort—and contrasting these with control samples—disease-specific pathway alterations were identified. Furthermore, SCI samples were stratified into high- and low-*CASP1* expression subsets to assess variations in the local immune microenvironment. Immune cell activities were quantified through single-sample Gene Set Enrichment Analysis (ssGSEA), and inter-group differences were evaluated via the Wilcoxon rank-sum test.

### 2.11 Establishment of rat SCI model and experimental grouping

A total of 36 rats were randomly allocated (computer-generated random numbers) into three equal groups (*n* = 12): a Sham group (T10 laminectomy only), a Saline group (SCI modeling followed by normal saline gavage), and a PCB2 group (SCI modeling followed by PCB2 gavage). To induce SCI, anesthetized rats (2%−3% isoflurane in 100% oxygen) underwent a T10 laminectomy, exposing the thoracic spinal cord. A spinal cord impactor (RWD Life Science, Model E03363-001) dropped a 40 g metal rod from a height of 60 mm onto the T10 segment. Immediate hind limb retraction upon impact and subsequent delayed hind limb paralysis post-anesthesia confirmed successful contusion. For 3 days post-surgery, all rats received prophylactic penicillin injections (40,000 U/kg for 3 days) and had unrestricted access to food and water.

Starting immediately after the contusion, rats in the PCB2 group were given PCB2 (50 mg/kg/day, dissolved in 10% DMSO and diluted with normal saline (final DMSO < 1%) via gentle vortexing) via oral gavage for seven consecutive days. The Saline group received an equivalent volume of 0.9% normal saline. Rats in the Sham group underwent the surgical exposure without the impact injury. Manual bladder expression was performed three times daily until autonomic voiding resume. While T10 laminectomy alone did not impair motor function in Sham rats, all injured rats initially recorded a score of 0 on the Basso, Beattie, and Bresnahan (BBB) scale, verifying complete hind limb paralysis.

### 2.12 Behavioral function evaluation

Hindlimb motor recovery was tracked using the BBB locomotor rating scale. Assessments were conducted on postoperative day 1 and then weekly over an 8-week period. Two independent investigators, blinded to the group assignments, observed each rat for 5 minutes. Scores spanned from 0 (total paralysis) to 21 (normal gait). The time taken for rats to regain spontaneous urination was also monitored as a measure of autonomic functional recovery. All behavioral, histological, and molecular assessments were performed by investigators blinded to group allocation.

### 2.13 Histopathological and immunofluorescence staining

At the 8-week endpoint, rats were deeply anesthetized with sodium pentobarbital (50 mg/kg, i.p.) and transcardially perfused with normal saline followed by 4% paraformaldehyde (PFA). A 2 cm spinal cord segment encompassing the lesion epicenter was excised, post-fixed in 4% PFA, and embedded in paraffin. Transverse sections (4 μm thick) were prepared and subjected to hematoxylin-eosin (H&E), Luxol fast blue (LFB), and Nissl staining. The slides were subsequently evaluated under a microscope scanner.

For immunofluorescence, the sections were deparaffinized, rehydrated, and subjected to antigen retrieval in sodium citrate buffer (pH 6.0). After blocking with 5% goat serum for 1 h at room temperature, sections were incubated overnight at 4 °C with an anti-*CASP1* primary antibody (1:200). An Alexa Fluor 594-conjugated secondary antibody (1:500) was applied the following day for 1 h at 37 °C. Nuclei were counterstained with DAPI for 5 min, and images were captured using a laser confocal microscope (Zeiss LSM 880, Germany).

### 2.14 Western blot analysis

Spinal cord tissue from the injury epicenter was homogenized in RIPA lysis buffer containing protease and phosphatase inhibitors. Protein concentrations were determined with a BCA assay kit (Beyotime, China). Equal amounts of protein (50 μg) were resolved on 12% SDS-PAGE gels and transferred to PVDF membranes at 250 mA for 90 min. Following a 1.5-h block in 5% non-fat milk, membranes were incubated overnight at 4 °C with primary antibodies against cleaved *CASP1* p10/p12 (1:1000) (~10 kDa p10 subunit) and GAPDH (1:5,000). After washing with TBST containing 0.1% Tween-20, an HRP-conjugated secondary antibody was applied for 1 h at room temperature. Protein bands were detected using an ECL chemiluminescence kit (Thermo Fisher Scientific, USA) and captured on a Tanon-5200 imaging system (China). The intensities of the cleaved CASP1 fragments were quantified using ImageJ (n = 3 biological replicates), with GAPDH serving as the loading control.

### 2.15 Statistical analysis

Data processing and statistical testing were performed using R (v4.2.3) and GraphPad Prism (v9.5.1). Continuous variables are reported as the mean ± standard error of the mean (SEM). Differences between two groups were analyzed via an unpaired *t*-test, whereas multi-group comparisons were conducted using Linear Mixed-Effects Model (LMM) with Bonferroni correction. A *P*-value < 0.05 was considered statistically significant. For histological and immunofluorescence data, multi-group comparisons were evaluated using One-way ANOVA followed by Tukey's *post-hoc* test.

## 3 Results

### 3.1 Identification of procyanidin B2 target genes

**Figure 2:**
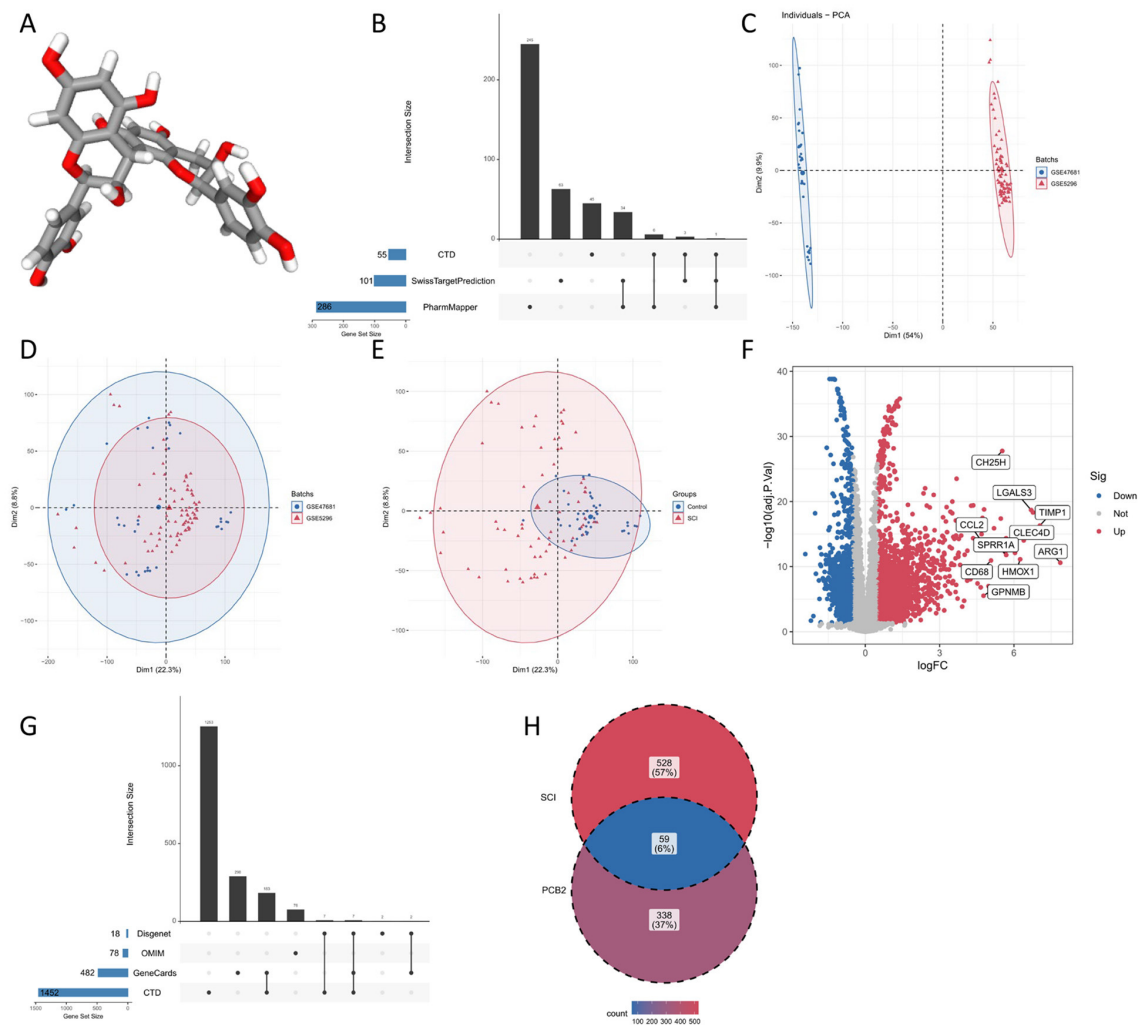
Identification of PCB2 targets and screening of SCI-related genes. **(A)** The 3D chemical structure of PCB2 retrieved from PubChem. **(B)** UpSet plot displaying the intersection of potential PCB2 targets predicted by PharmMapper, SwissTargetPrediction, and CTD databases. **(C)** Principal component analysis (PCA) plot of GSE5296 and GSE47681 datasets before batch correction, showing significant batch effects. **(D)** PCA plot after batch correction, demonstrating effective removal of non-biological variations. **(E)** PCA plot showing clear separation between SCI and control samples after data integration. **(F)** Volcano plot of DEGs between SCI and sham groups. Red dots represent upregulated genes, and blue dots represent downregulated genes. **(G)** UpSet plot illustrating the overlap of SCI-related genes obtained from DisGeNET, OMIM, GeneCards, and CTD databases. **(H)** Venn diagram showing the intersection of 59 common targets shared between PCB2-predicted targets and SCI-related genes.

The 3D molecular structure of PCB2 was acquired from the PubChem database (Figure 2A). By querying PharmMapper, SwissTargetPrediction, and the CTD database, its potential biological targets were mapped out. Merging these datasets and filtering out duplicate records yielded a final set of 397 unique candidate targets for PCB2 (Figure 2B).

### 3.2 Identification of SCI-related target genes

To construct a robust SCI expression profile, the GSE5296 and GSE47681 microarray datasets from GEO were merged and the expression matrices for both injured and sham tissues were normalized. Post-normalization PCA plots (Figures 2C, D) confirmed the mitigation of batch effects, showing a distinct separation between SCI and control cohorts (Figure 2E). Applying a threshold of |log2 fold change| ≥ 0.5 and adjusted *P*-value ≤ 0.05 in the limma analysis, 3,939 differentially expressed genes (DEGs)-−1,812 upregulated and 2,127 downregulated in the injured state—were extracted. These expression shifts are visualized in volcano plots (Figure 2F).

A comprehensive SCI gene pool was simultaneously built by mining GeneCards (relevance score ≥ 1), OMIM, CTD (inference score ≥ 20), and DisGeNET database with the keyword “spinal cord injury.” Integrating these sources and removing duplicates generated a list of 1,882 unique SCI-associated genes (Figure 2G).

### 3.3 PPI network and functional enrichment analysis of candidate targets

**Figure 3:**
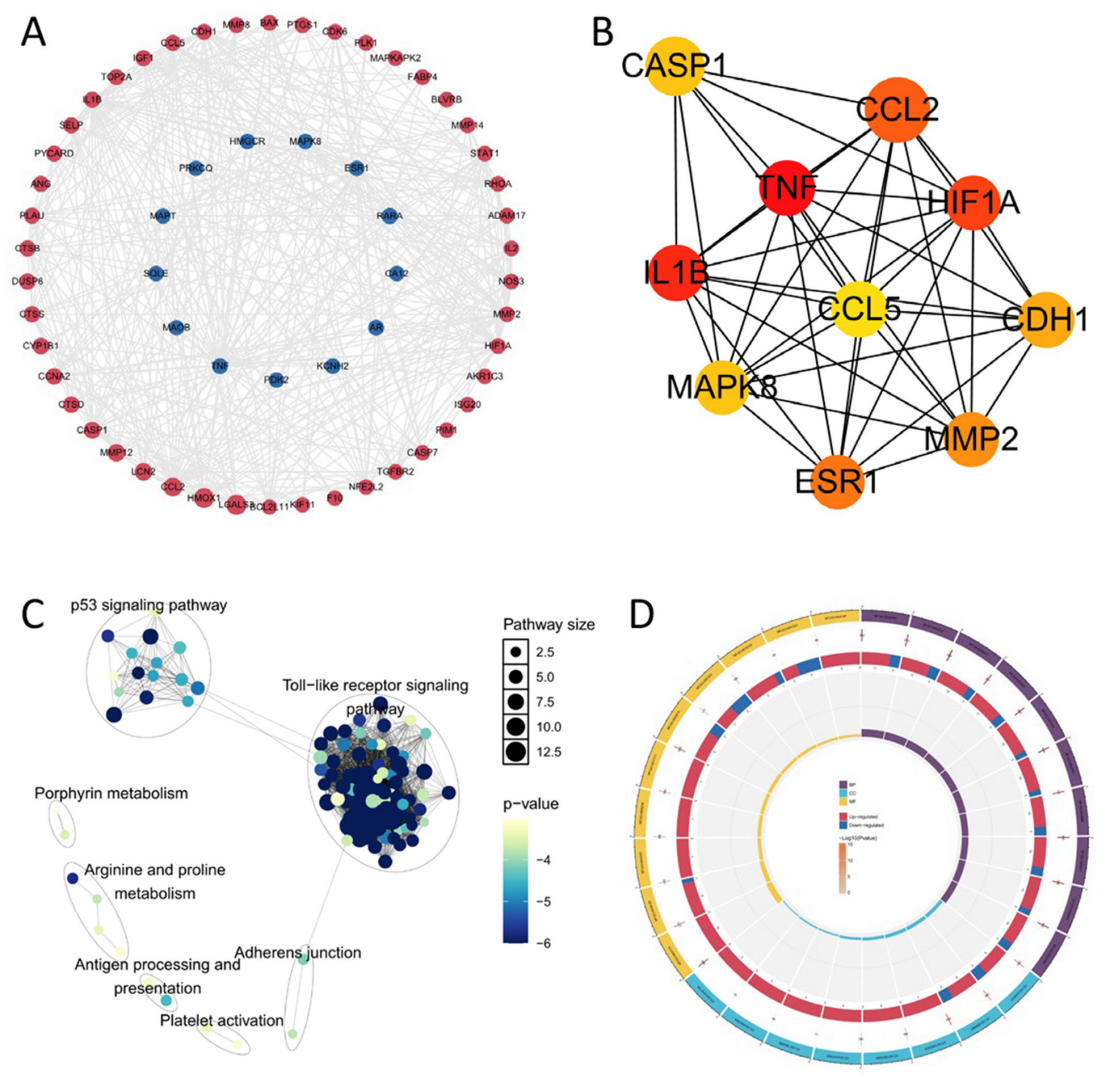
PPI network construction and functional enrichment analysis of candidate targets. **(A)** The PPI network of 59 intersecting targets constructed using the STRING database and visualized via Cytoscape. Nodes represent proteins, and edges represent interactions. **(B)** The top 10 hub genes (TNF, IL1B, HIF1A, CCL2, MMP2, ESR1, CCL5, CDH1, MAPK8, and *CASP1*) identified by the CytoHubba plugin using the Degree method. **(C)** GO enrichment analysis of the candidate genes, including BP, CC, and MF. **(D)** KEGG pathway enrichment analysis showing the top enriched signaling pathways associated with PCB2 treatment in SCI.

Intersecting the DEGs from the transcriptomic analysis with the database-derived SCI genes resulted in 587 genes closely linked to the pathology. When this set was mapped against the 397 predicted PCB2 targets, 59 common genes emerged as the core candidates for PCB2-mediated therapy (Figure 2H; Supplementary Table S1). The protein-protein interactions (PPI) among these 59 genes were then modeled using the STRING database and Cytoscape (Figure 3A). Degree-based topological analysis via CytoHubba highlighted the top 10 hub genes: *TNF, IL1B, HIF1A, CCL2, MMP2, ESR1, CCL5, CDH1, MAPK8*, and *CASP1*, emphasizing their structural importance in the network (Figure 3B).

To decipher the biological functions of these 59 targets, GO and KEGG pathway enrichment analyses were conducted (Figures 3C, D; Supplementary Tables S2, S3).

KEGG pathway profiling pointed to several critical signaling cascades involved in neuroprotection and secondary injury. Prominent pathways included Toll-like receptor signaling, a primary driver of glial inflammation and innate immunity (34); p53 signaling, which regulates oxidative stress and apoptosis (35, 36); and adherens junction assembly, which is essential for blood-spinal cord barrier integrity. Other enriched terms, such as platelet activation and antigen processing, underscored the roles of hemostasis and leukocyte recruitment during the acute injury phase.

GO biological process analysis revealed a strong focus on inflammatory and regenerative events, such as responses to lipopolysaccharides, apoptotic modulation, and wound healing (37, 38). In terms of cellular components, the targets were primarily localized to membrane rafts, microdomains, and collagen-rich extracellular matrices—structures vital for maintaining tissue architecture during post-injury remodeling (39, 40). Molecular function terms highlighted cytokine receptor binding and various peptidase activities, pointing to the regulation of inflammatory cytokine networks and extracellular matrix degradation (41, 42). These enrichment patterns suggest that PCB2 likely confers neuroprotection by stabilizing tissue structure, curbing apoptosis, and resolving inflammation.

### 3.4 Machine learning-based identification of core regulators

**Figure 4:**
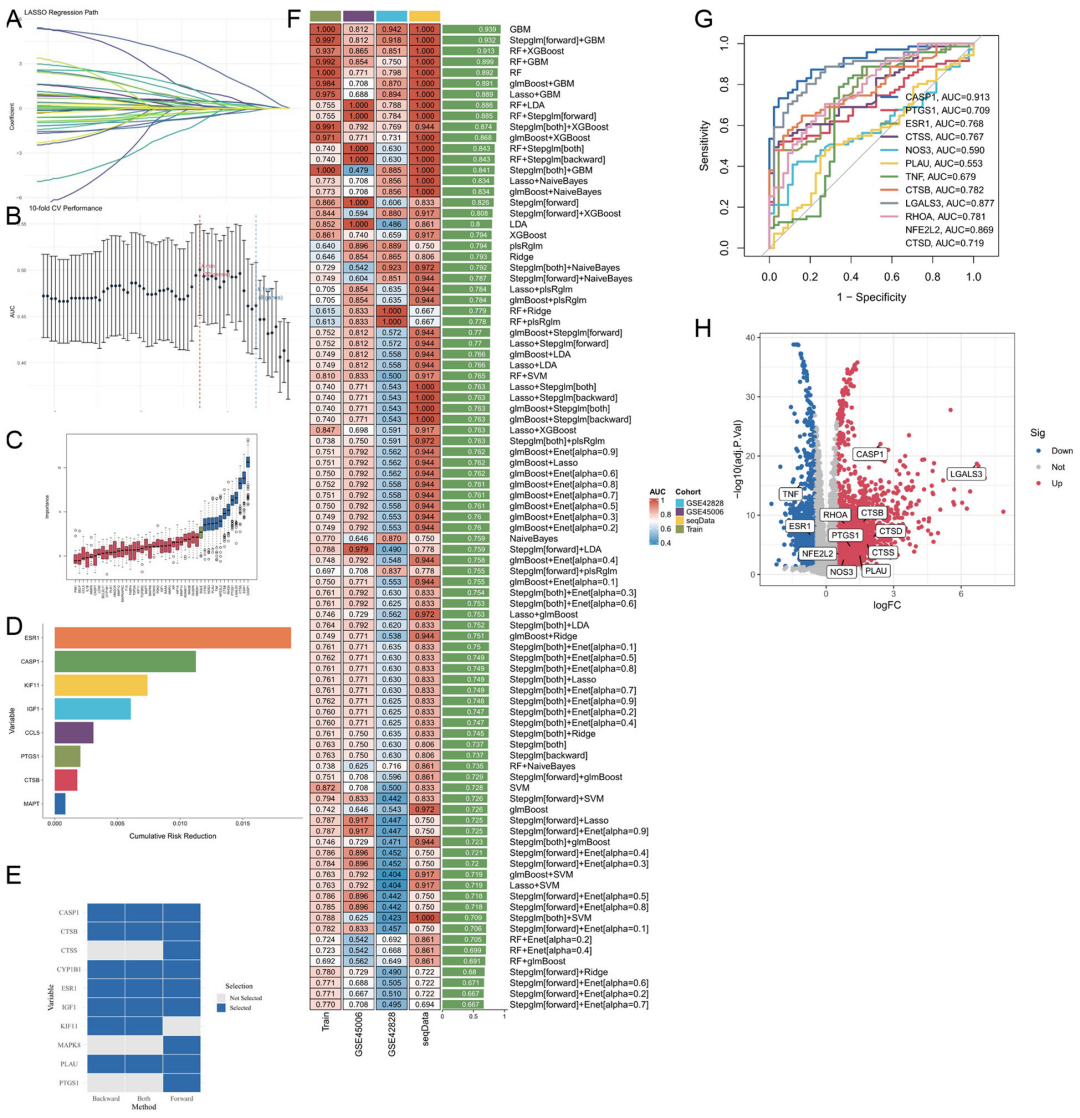
Identification and Validation of Core Potential Targets for PCB2 in SCI via Integrated Machine Learning Approaches. **(A)** Coefficient profile plot of the LASSO regression model showing the log(lambda) sequence. **(B)** Partial likelihood deviance plot for the LASSO regression, identifying 8 candidate genes at the optimal lambda. **(C)** Feature importance ranking of candidate diagnostic genes derived from the Random Forest algorithm. **(D)** Bar chart displaying the importance of the top variables identified by the Gradient Boosting algorithm. **(E)** Heatmap illustrating the selection of optimal gene signatures (blue indicates selected) across three Stepwise regression strategies: Backward, Forward, and Both (bidirectional). **(F)** Comprehensive performance evaluation of various machine learning models and their ensemble combinations. The heatmap visualizes the Area Under the Curve (AUC) values across the training set and three validation cohorts. **(G)** ROC curves evaluating the diagnostic efficacy of the identified core genes in distinguishing SCI samples from controls. **(H)** Volcano plot illustrating the differential expression trends of the machine learning-derived key genes.

To identify the most critical regulatory genes governing the therapeutic response to PCB2, we evaluated the 59 hub genes using a multi-algorithm machine learning approach. Variable screening was performed via LASSO regression, Glmboost, stepwise regression variants, and random forest. LASSO regression narrowed the list to 8 candidate genes (*CASP1*, PTGS1, IGF1, CCL5, KIF11, CTSB, ESR1, MAPT) (Figures 4A, B), while random forest prioritized the top 12 targets (Figure 4C). Glmboost also converged on a set of 8 genes (Figures 4D), and backward, combined, and forward stepwise regression isolated 7, 7, and 9 genes, respectively (Figure 4E). Building upon these results, we constructed ensemble classifiers to pinpoint the dominant regulators. A combined RF-XGBoost model outperformed the others, achieving an AUC of 0.937 on the training cohort and demonstrating robust generalization across three validation sets (AUCs: 0.865, 0.851, 1.000). The overall validation AUC of 0.913 confirmed the model's high predictive accuracy (Figure 4F).

Feature importance analysis extracted from this hybrid model ranked 12 key genes—*CASP1, PTGS1, ESR1, CTSS, NOS3, PLAU, TNF, CTSB, LGALS3, RHOA, NFE2L2*, and *CTSD*—as the primary discriminators between SCI and normal tissues. ROC analysis further validated their diagnostic value; *CASP1* alone achieved an AUC of 0.913, marking it as a strong standalone biomarker for SCI pathology (Figure 4G). Consistent with these findings, the combined GSE5296 and GSE47681 dataset revealed that neuroprotective factors like NFE2L2 were suppressed after SCI, whereas pro-inflammatory and pro-apoptotic drivers like *CASP1* were significantly upregulated (Figure 4H).

**Figure 5:**
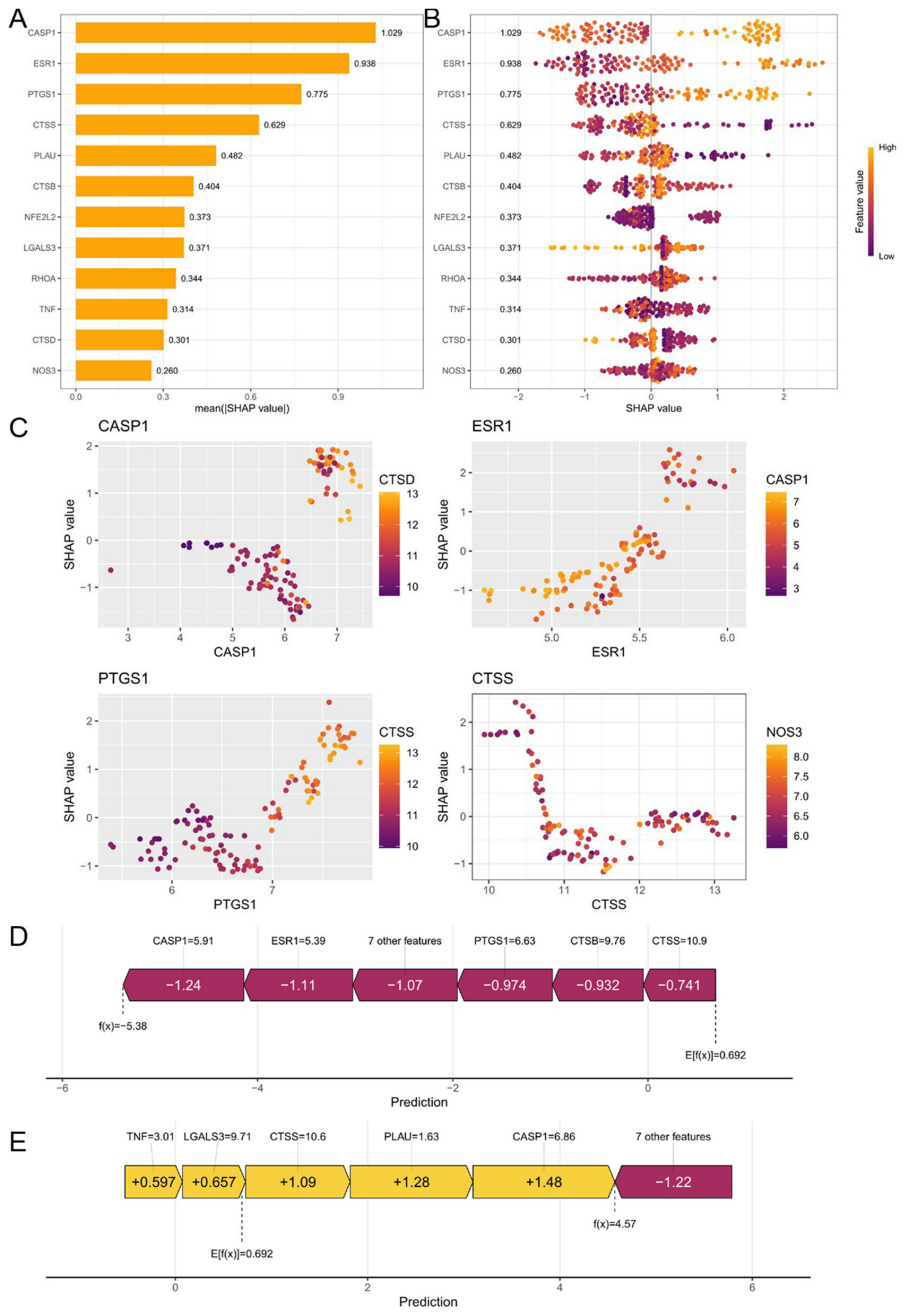
Interpretability Analysis of the SCI diagnostic model using SHAP. **(A)** Bar chart illustrating the global feature importance of the top predictive genes, ranked by the mean absolute SHAP values. **(B)** SHAP summary plot displaying the distribution of SHAP values for each gene across all samples. Each dot represents a sample; color indicates the gene expression level (purple for low, yellow for high). Points to the right of the zero line indicate a positive contribution to the SCI risk prediction, while points to the left indicate a negative contribution. **(C)** SHAP dependence plots for key genes. **(D)** A control (Sham) sample with a low predicted risk score [*f* (*x*) = −5.38], where low expression of *CASP1* (−1.24) and ESR1 (−1.11) contributes to the negative prediction. **(E)** An SCI sample with a high predicted risk score [*f* (*x*) = 4.57], primarily driven by the positive contributions of high *CASP1* (+1.48) and PLAU (+1.28) expression.

To make the model interpretable, SHAP values for each feature were calculated. *CASP1* (SHAP value: 1.029) and PTGS1 (0.775) emerged as the strongest predictive drivers, followed by ESR1 (0.0938) and related downstream molecules (Figures 5A, B). Additionally, SHAP dependence analysis uncovered a linear positive correlation between ESR1 expression and the model's risk output, as well as a marked synergistic interaction between ESR1 and *CASP1* (Figure 5C). For instance, a control (Sham) sample yielded a strongly negative score (−5.38), driven by low levels of *CASP1* (−1.24) and ESR1 (−1.11), correctly predicting the uninjured state (Figure 5D). Conversely, in a representative SCI sample, the model output a high-risk score of 4.57 (base value: 0.692), which was heavily influenced by the upregulation of *CASP1* (+1.48) and PLAU (+1.28) (Figure 5E).

### 3.5 Single-cell transcriptomic profiling of CASP1 expression dynamics

**Figure 6:**
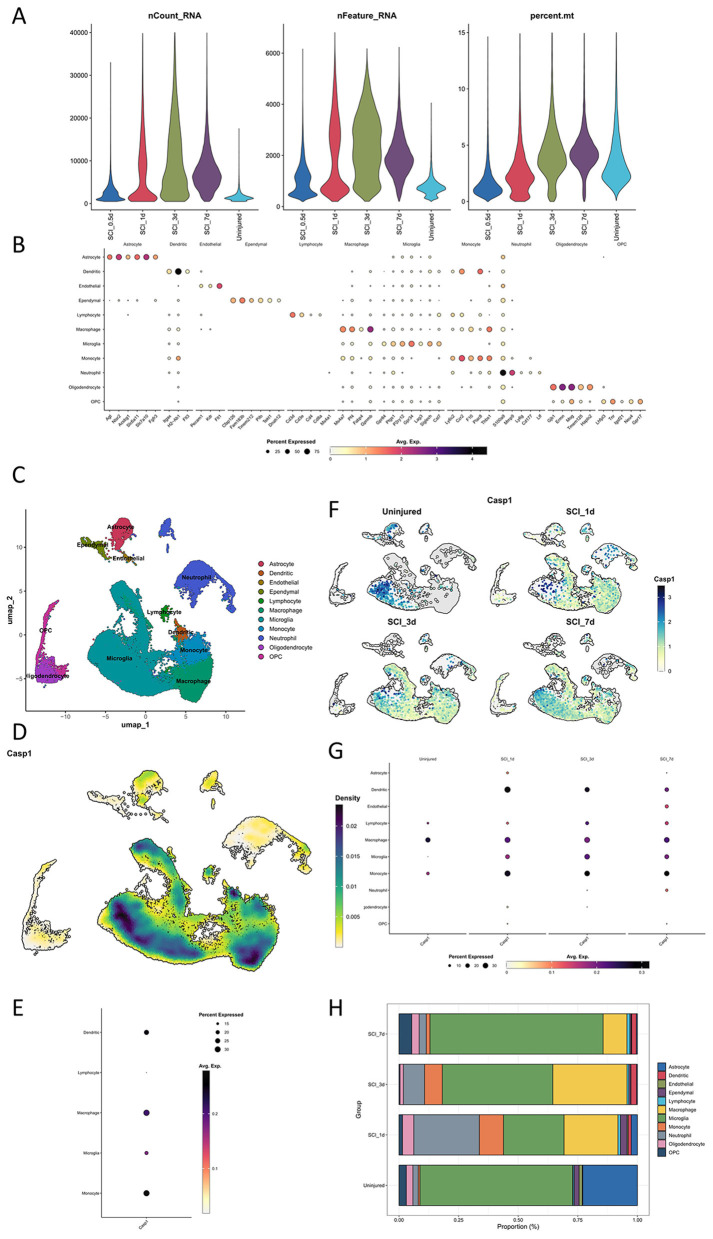
Single-cell transcriptomic profiling reveals the cellular landscape and *CASP1* expression dynamics in SCI. **(A)** Quality control metrics for the scRNA-seq dataset. **(B)** Dot plot showing the expression of canonical marker genes used to annotate cell clusters. The size of the dot represents the percentage of cells expressing the marker, and color intensity indicates the average expression level. **(C)** Uniform manifold approximation and projection (UMAP) visualization of 11 distinct cell clusters identified in the spinal cord tissue. **(D)** Feature plot illustrating the specific distribution of *CASP1* expression across all cell clusters. High expression levels (blue) are predominantly localized to myeloid lineage cells. **(E)** Dot plot confirming the specific enrichment of *CASP1* in the cell types. **(F)** Split UMAP plots displaying the temporal dynamics of *CASP1* expression following injury. **(G)** Dot plot quantifying the temporal changes in *CASP1* expression intensity and frequency within cell populations across the different time points. **(H)** Stacked bar chart showing the relative proportions of each cell type across the different experimental groups.

To probe the cellular dynamics of *CASP1* and assess PCB2′s capacity to modulate inflammation, the scRNA-seq dataset GSE189070 was analyzed. Following rigorous quality control filtering (Figure 6A), the cells were clustered into 11 distinct neural and immune populations (Figures 6B, C). Uniform Manifold Approximation and Projection (UMAP) projections revealed that *CASP1* expression was primarily restricted to myeloid cells, such as macrophages, microglia, monocytes, and dendritic cells (Figures 6D, E). While *CASP1* was barely detectable in uninjured controls, it became sharply upregulated across these myeloid populations after SCI (Figures 6F, G). This expression shift points to *CASP1* as a core mediator of the injury-induced inflammatory cascade within the innate immune system. Concurrently, the SCI cohort exhibited a significant expansion of neutrophils and macrophages compared to controls (Figure 6H), confirming a state of intense local immune activation and framing this inflammatory microenvironment as the primary target for PCB2 therapy.

### 3.6 Molecular docking of PCB2 and CASP1-mediated functional networks

**Figure 7:**
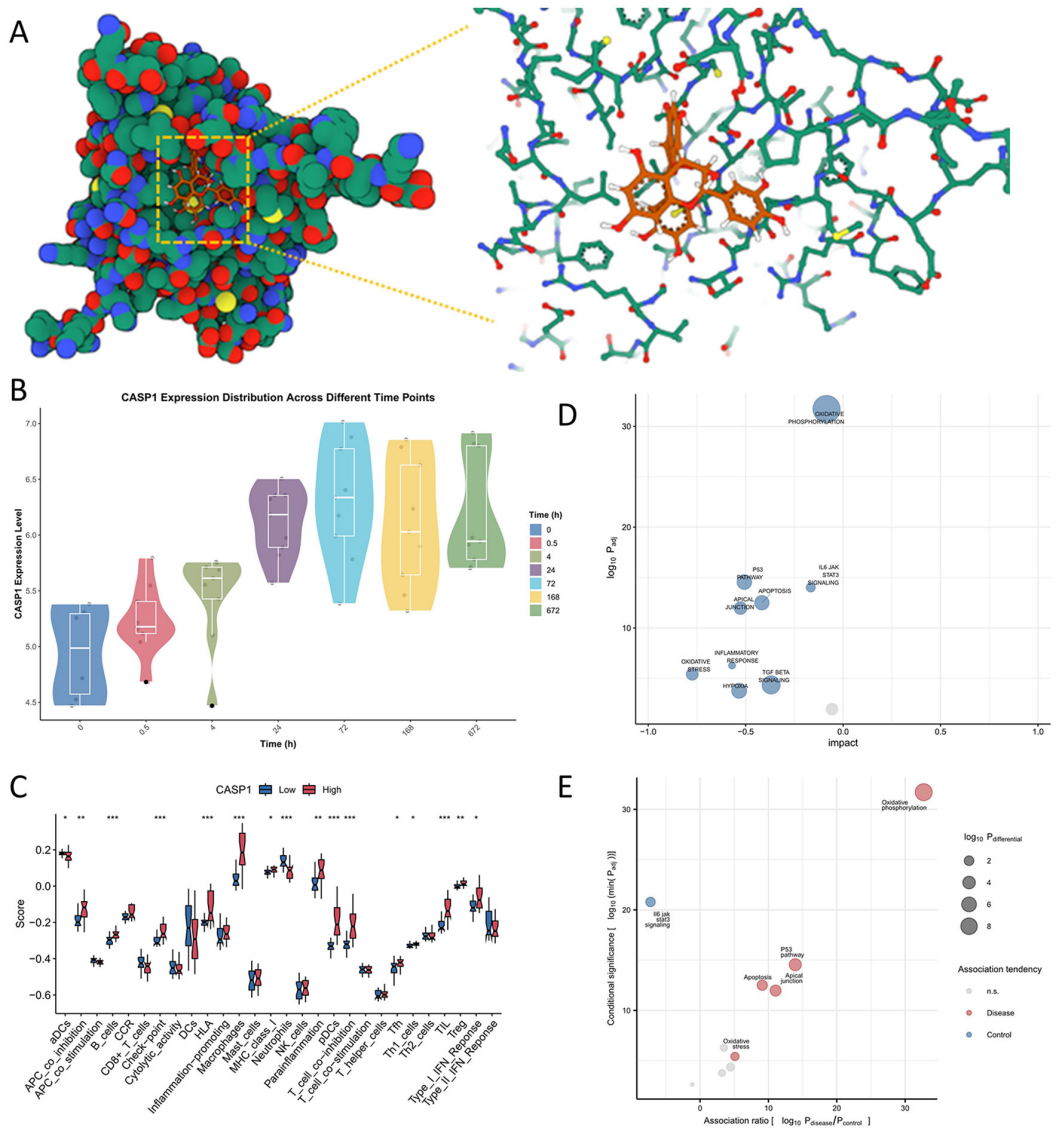
Molecular interaction landscape and functional network analysis of the core target *CASP1*. **(A)** Molecular docking simulation of PCB2 with the *CASP1* protein. **(B)** Temporal expression dynamics of *CASP1* in SCI tissue. **(C)** Analysis of immunological functional differences in the SCI microenvironment stratified by *CASP1* expression. **(D)** Volcano plot illustrating the functional co-expression signature of *CASP1* within the SCI group. The x-axis represents the impact (strength and direction of co-expression), and the y-axis indicates the statistical significance (–log10 adjusted *P*-value) of the association between *CASP1* and specific pathways. **(E)** Differential co-expression analysis comparing the *CASP1* functional network between SCI and Sham groups. Bubble size corresponds to the statistical significance of the difference. Red bubbles (positive values) denote pathways more strongly associated with *CASP1* in the SCI group, whereas blue bubbles (negative values) indicate pathways with stronger *CASP1* associations in the control group.

Computational docking simulations were employed to predict the direct interaction between PCB2 and *CASP1*. The analysis yielded a highly favorable docking score of −7.2 kcal/mol, suggesting that PCB2 stably binds to the *CASP1* active site and may act as a direct inhibitor (Figure 7A). In parallel, transcriptome data from GSE5296 confirmed that *CASP1* transcription remained consistently elevated throughout the first 72 h post-injury (Figure 7B), marking it as a sustained driver of acute inflammation.

To understand the broader immunological impact of *CASP1*, the SCI samples were divided into high- and low-expression groups based on the median *CASP1* level. The high-*CASP1* group displayed significant enrichment in multiple immune pathways, including B cell and macrophage activation, T-cell co-inhibition, regulatory T cells, and parainflammation (Figure 7C). These shifts indicate that excessive *CASP1* expression exacerbates the pro-inflammatory milieu following spinal cord trauma.

The functional network of *CASP1* was further explored using GeneCOCOA comparative co-expression analysis. Within the SCI cohort, *CASP1* exhibited strong correlations with metabolic and cell-death signatures, most notably Oxidative Phosphorylation and the P53 pathway (Figure 7D). It also co-expressed with genes governing Apoptosis and Apical Junction dynamics. When contrasted against control samples, this network revealed profound disease-specific rewiring (Figure 7E). The coupling of *CASP1* with Oxidative Phosphorylation, Apoptosis, and P53 pathways was almost exclusively observed in the injured state, rather than being a baseline homeostatic function. In contrast, pathways like KRAS signaling lacked this disease-specific association. Together, these data suggest that SCI triggers a pathological repurposing of *CASP1*, linking it directly to mitochondrial stress and programmed cell death (43, 44).

### 3.7 Experimental validation of PCB2 for SCI recovery

**Figure 8:**
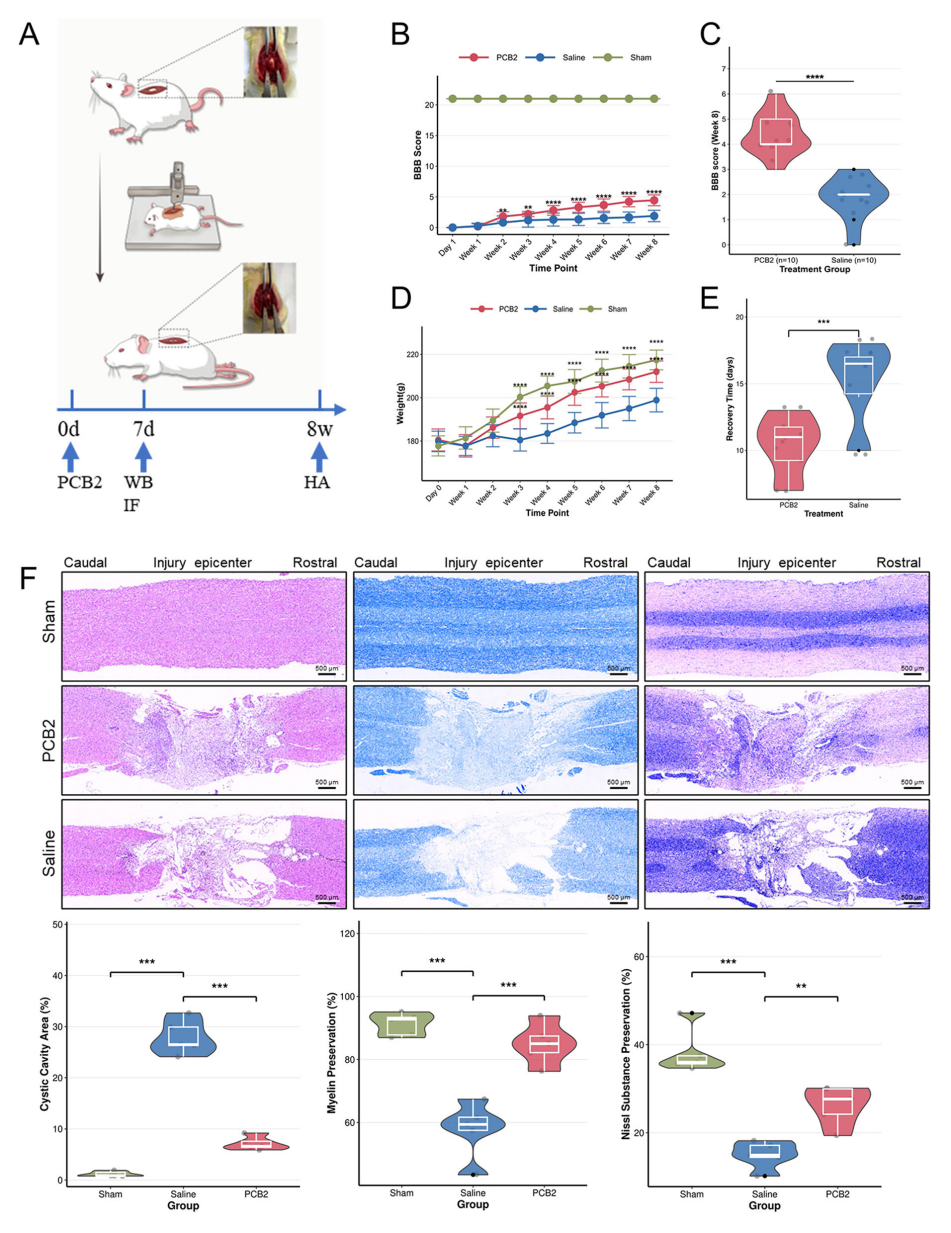
Experimental validation of the therapeutic efficacy of PCB2 in a rat SCI model. **(A)** Schematic illustration of the experimental design and timeline. **(B)** BBB locomotor scores for Sham, Saline, and PCB2 groups evaluated from day 1 to week 8 post-surgery. **(C)** Violin plot showing the distribution of BBB scores at week 8. **(D)** Postoperative body weight changes of rats in each group over the 8-week observation period. **(E)** Quantification of the recovery time for autonomous urination function. **(F)** Representative histological images of spinal cord sections at the injury epicenter and adjacent regions (rostral/caudal) at 8 weeks post-injury, including sections stained with H&E staining, LFB and Nissl staining. *p < 0.01, **p < 0.001, ***p < 0.0001.

The therapeutic efficacy of PCB2 was evaluated *in vivo* through assessments of body weight, urination, motor recovery, and tissue pathology (Figure 8A). BBB locomotor tracking over 8 weeks showed that while all injured rats suffered immediate and near-complete hindlimb paralysis, the PCB2-treated group exhibited significantly faster and more robust functional recovery starting from the second week (Figures 8B, C, Supplementary Table S4). PCB2 treatment also accelerated the return to preoperative body weight (Figure 8D) and significantly shortened the latency to regain autonomous urination (Figure 8E), reflecting broad systemic and autonomic benefits.

**Figure 9:**
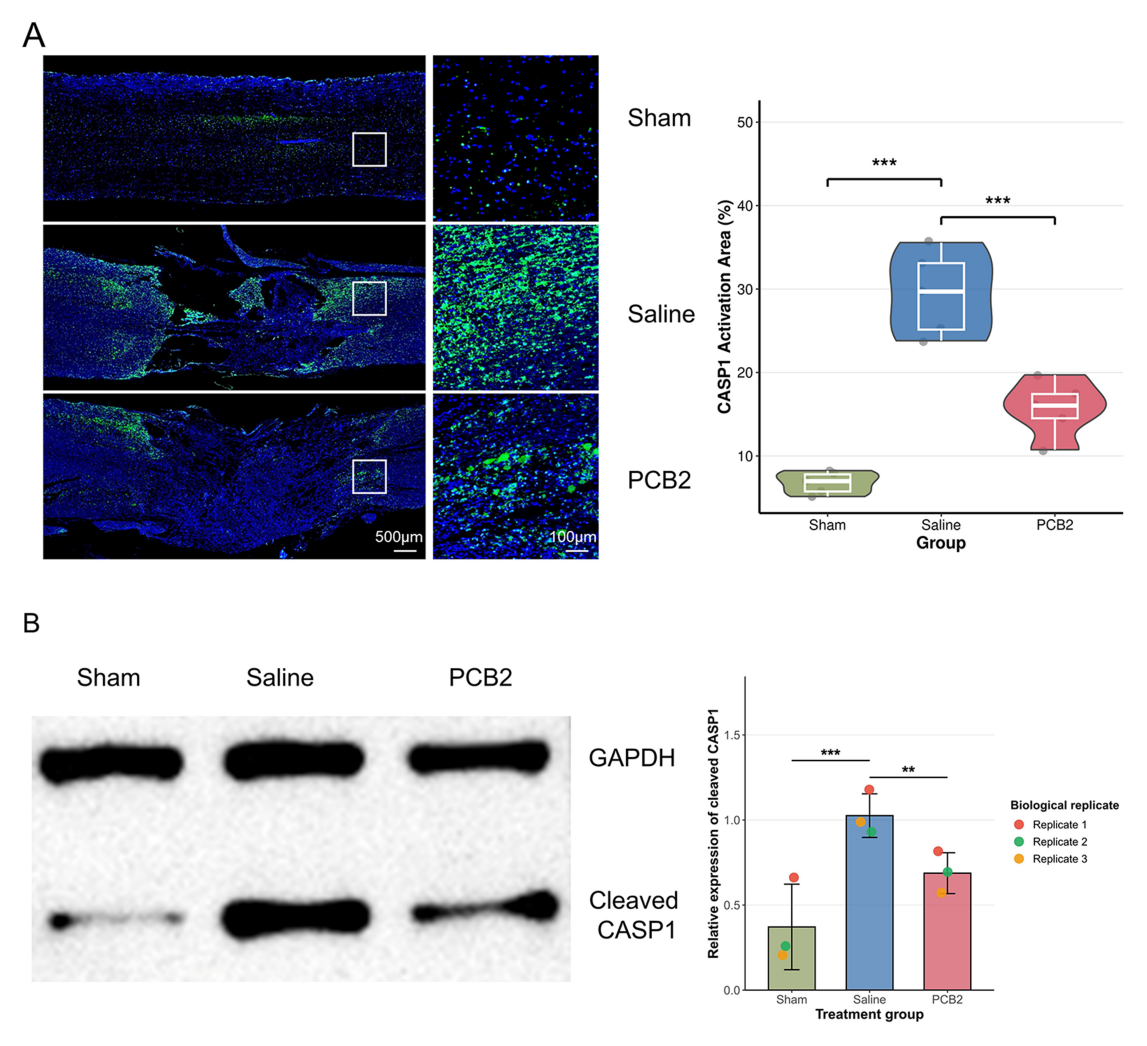
Experimental validation of the effect of PCB2 on CASP1 expression in a rat SCI model. **(A)** Representative immunofluorescence staining images of CASP1 in the injured spinal cord tissues. **(B)** The Western blot bands showing the protein expression levels of CASP1 and GAPDH (internal control) in spinal cord tissues from Sham, Saline, and PCB2 groups, and quantitative analysis of relative CASP1 protein expression normalized to GAPDH. *p < 0.05, **p < 0.01, ***p < 0.001, ****p < 0.0001.

Histological examinations at week 8 corroborated these functional improvements. Untreated SCI rats displayed extensive cystic cavities, severe neuronal and myelin loss, and dense inflammatory infiltrates (Figure 8F). PCB2 intervention markedly reduced the lesion volume, preserved neuronal architecture and myelin sheaths, and attenuated local inflammation. At the molecular level, immunofluorescence staining showed far fewer CASP1-positive cells in the PCB2 group than in the SCI controls (Figure 9A). Western blot analysis confirmed this trend: the injury-induced spike in CASP1 protein levels was strongly suppressed by PCB2 administration (Figure 9B). These combined *in vivo* results validate our bioinformatic predictions, demonstrating that PCB2 promotes neuroprotection and tissue repair largely by suppressing CASP1 expression, while docking models suggest it also has the potential to interfere with downstream inflammatory pathways.

## 4 Discussion

Secondary injury cascades remain the primary barrier to functional recovery after SCI, even with recent advances in surgical and rehabilitative care. We explored the neuroprotective potential of Procyanidin B2 (PCB2) in the context of SCI. The principal finding of our work is that PCB2 delivers substantial therapeutic benefits, notably driving motor recovery and minimizing tissue destruction, largely by interrupting the CASP1-driven inflammatory cascade. Using a blend of transcriptomics, machine learning, and animal models, we isolated CASP1 as the primary therapeutic target. Our data suggest a potential mechanism: *in vivo*, PCB2 markedly suppresses the injury-induced spike in cleaved CASP1 fragments, while docking studies suggest it may also potentially bind to the enzyme's active site. By simultaneously downregulating CASP1 activation and potentially interfering with its catalytic pocket, PCB2 effectively cools the hostile inflammatory microenvironment at the lesion core, halting the progression of secondary damage and fostering tissue repair.

The robust phenotypic improvements in our rat contusion model clearly show the therapeutic efficacy of PCB2. After severe thoracic SCI, the acute inflammatory response usually drives rapid tissue cavitation and extensive demyelination. This sequence leads to persistent motor paralysis and autonomic dysfunction (2, 45). Our longitudinal behavioral tracking showed that PCB2 administration accelerated functional recovery. Treated rats had higher Basso, Beattie, and Bresnahan (BBB) locomotor scores, indicating better hindlimb coordination and weight-bearing capacity than untreated controls. PCB2 also sped up the return of spontaneous urination, an autonomic milestone that profound secondary cord damage often delay (46). These functional gains matched the tissue-level preservation. Histological and immunofluorescence analyses confirmed that PCB2 restricted cystic cavity expansion, preserved myelin architecture, and reduced local inflammatory infiltration. These *in vivo* data provide evidence that PCB2 acts as a potent neuroprotective agent, rescuing spinal cord tissue from secondary injury rather than functioning solely as a generic antioxidant.

To explain these phenotypic improvements, we focused on the inflammasome-effector enzyme *CASP1* and its role in the local immune microenvironment. Mechanical disruption of the blood-spinal cord barrier during SCI triggers a massive influx of peripheral immune cells. These infiltrating cells, along with resident microglia, drive a severe neuroinflammatory cascade (4). Our single-cell RNA sequencing (scRNA-seq) analysis offered necessary spatial and cellular context, showing that the pathological upregulation of *CASP1* does not occur uniformly across the tissue. Instead, it localizes specifically to infiltrating macrophages and activated microglia. Within these myeloid populations, *CASP1* functions as the primary executioner of the inflammasome pathway. Its activation drives the maturation and release of pro-inflammatory cytokines, such as IL-1β and IL-18, and triggers gasdermin D-mediated neuronal pyroptosis (18, 47). Locating *CASP1* in this specific myeloid niche identifies the exact cellular arena for PCB2 action. By suppressing *CASP1* expression and likely inhibiting its enzymatic activity directly within these immune sentinels, PCB2 neutralizes the main source of the inflammatory cytokine storm. This prevents subsequent pyroptotic cell death and shifts the local microenvironment from a neurotoxic to a neuroprotective state.

Beyond targeting the inflammasome, our bioinformatics and comparative co-expression analyses revealed the broader pathological network that *CASP1* orchestrates during SCI. Using the GeneCOCOA framework to assess context-specific gene co-expression, we found a distinct disease-specific rewiring of the *CASP1* functional network. *CASP1* shows minimal interaction with metabolic or cell-death pathways in the healthy spinal cord. Following SCI, however, its expression tightly couples with oxidative phosphorylation and the p53 signaling cascade. This metabolic and apoptotic reprogramming indicates that *CASP1* activation acts as a central hub—rather than an isolated inflammatory event—that exacerbates mitochondrial stress, increases reactive oxygen species (ROS) production, and accelerates programmed cell death (36, 44). Concurrently, KEGG enrichment analysis of PCB2 candidate targets identified the Toll-like receptor signaling pathway, a primary upstream initiator of glial inflammation that converges on *CASP1* activation (34). Furthermore, complementary neuroprotective mechanisms, such as the modulation of the JAK/STAT signaling axis by other polyphenols (48), may also act synergistically. These findings suggest PCB2 offers therapeutic benefits beyond direct *CASP1* inhibition. By neutralizing ROS and suppressing upstream innate immune receptors, PCB2 exerts a multi-level regulatory effect. This regulation uncouples *CASP1* from destructive metabolic and apoptotic networks, helping to restore cellular homeostasis and prevent further tissue degradation.

The closed-loop methodological design of this study strengthens the robustness of our findings. Traditional network pharmacology frequently encounters false-positive predictions because it relies on static, non-contextual databases and blind molecular docking (18). We addressed these limitations by integrating multi-algorithm machine learning with single-cell spatial transcriptomics and *in vivo* wet-lab validation. Our ensemble machine learning approach—combining Random Forest, XGBoost, and LASSO regression—provided precise dimensionality reduction. This filtered out background noise and isolated *CASP1* as the highest-weight diagnostic and therapeutic biomarker. Additionally, using scRNA-seq enabled us to look beyond bulk tissue averages and map the computational prediction directly to its functional cellular niche (macrophages and microglia). This integrated workflow confirms that the identified target is a biologically active, pharmacologically targetable node rather than a statistical artifact. This approach increases the reliability of our results and bridges the gap between theoretical predictions and translational applications.

This study has several limitations. First, while the rat thoracic contusion model mimics the mechanical forces of human SCI, inherent interspecies differences in spinal cord anatomy, immune responses, and neuroplasticity could affect translational outcomes, requiring cross-species and large animal validation. Second, although docking suggested potential binding, we assessed CASP1 expression rather than direct enzymatic activity; future assays are needed to confirm direct inhibition. Third, only CASP1 was experimentally validated *in vivo* among the computationally predicted targets (leaving the other 58 candidate targets unvalidated), and the scRNA-seq data contains biological noise. Fourth, the single oral dose (50 mg/kg/day) highlights the need for future dose-response and pharmacokinetic studies to optimize blood-spinal cord barrier penetrance, where nano-formulations could be explored. Finally, while we examined acute responses up to 8 weeks post-injury, chronic follow-ups are necessary to confirm whether PCB2 supports long-term neuroregeneration and durable functional recovery.

## 5 Conclusions

In summary, we utilized a combination of machine learning, network pharmacology, single-cell transcriptomics, and animal models to systematically elucidate the neuroprotective role of PCB2 in SCI. Phenotypically, PCB2 offers robust neuroprotection by driving motor recovery, shrinking lesion cavities, and sparing myelin. From a mechanistic standpoint, our network analyses suggest that PCB2 exerts a broad, multi-target influence over inflammation, apoptosis, and tissue remodeling. More specifically, we established that its core mechanism relies on targeting CASP1 within the macrophage and microglia population, where it blocks secondary inflammation by suppressing the expression of this enzyme. Ultimately, our findings further substantiate the role of CASP1 as a critical driver of the SCI pathological cascade and provide a strong rationale for advancing PCB2 as a viable, natural, multi-target candidate for future clinical translation.

## Data Availability

The original contributions presented in the study are included in the article/Supplementary material, further inquiries can be directed to the corresponding authors.

## Supplementary material

Supplementary Table S1 Complete list of 59 intersecting target genes between PCB2 potential targets and SCI-related genes.

Supplementary Table S2 Complete KEGG pathway enrichment analysis results for 59 candidate target genes.

Supplementary Table S3 Complete GO enrichment analysis results for 59 candidate target genes.

Supplementary Table S4 Individual BBB locomotor scores for all rats across different time points.
