# Supplementary material for: Neuroprotective potential of the natural polyphenol Procyanidin B2 in spinal cord injury: a comprehensive study utilizing machine learning, network pharmacology, and in vivo validation
Source: Front Nutr. 2026 Jul 6;13:1872188. doi: 10.3389/fnut.2026.1872188 (PMC13381209; doi:10.3389/fnut.2026.1872188)

# Additional file 1: Full uncropped western blots

This file contains full, uncropped western blot images supporting the blot panels in the manuscript.

| Replicate | Raw exposure (uncropped) | Marker |
| --- | --- | --- |
| 1 | R1_raw_exposure.tif | R1_marker.tif |
| 2 | R2_raw_exposure.tif | R2_marker.tif |
| 3 | R3_raw_exposure.tif | R3_marker.tif |

## Replicate 1

### Raw exposure (uncropped)


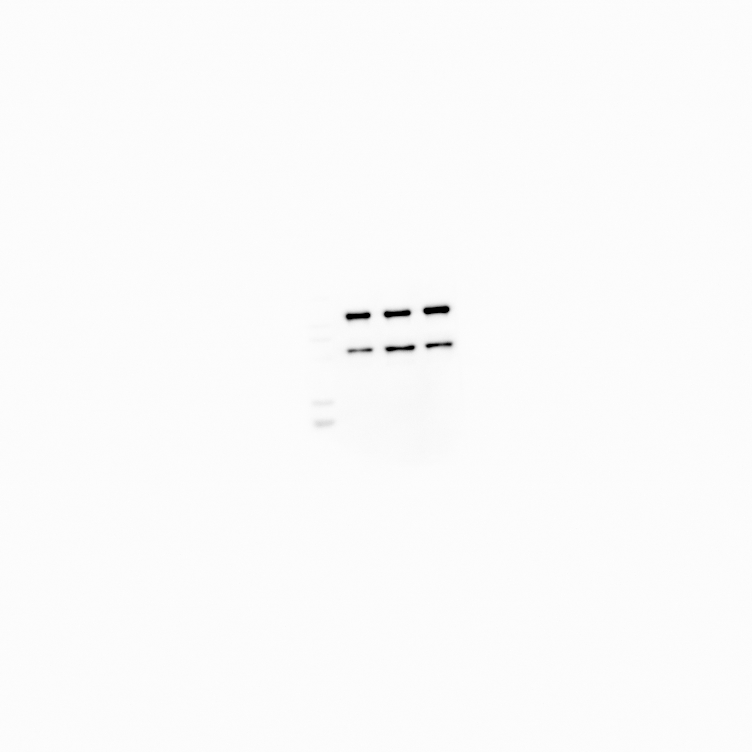


### Marker


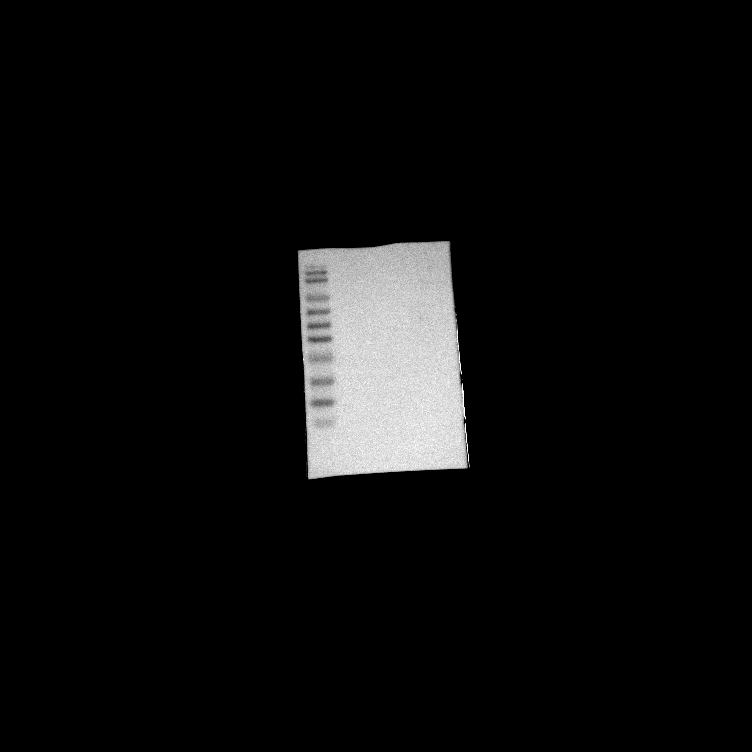


## Replicate 2

### Raw exposure (uncropped)


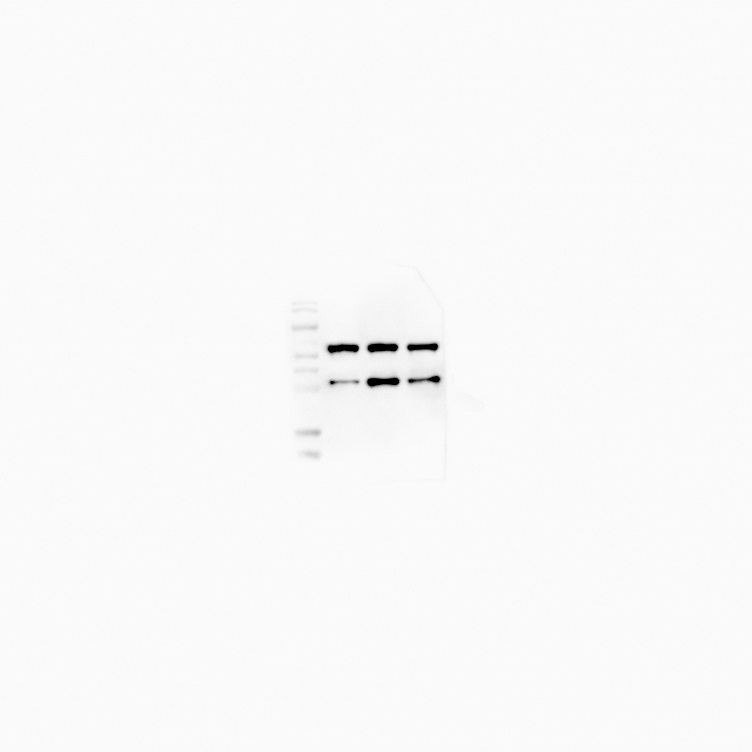


### Marker


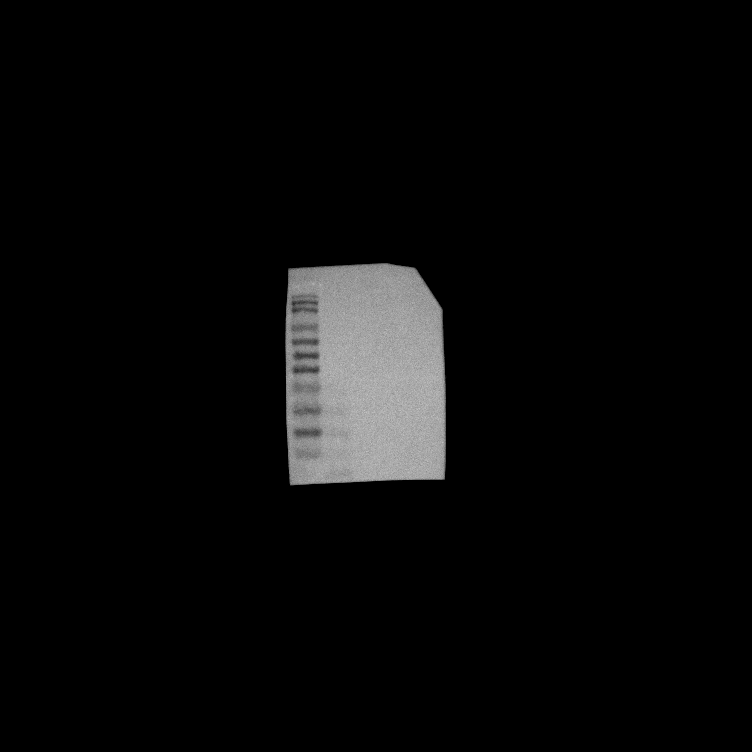


## Replicate 3

### Raw exposure (uncropped)


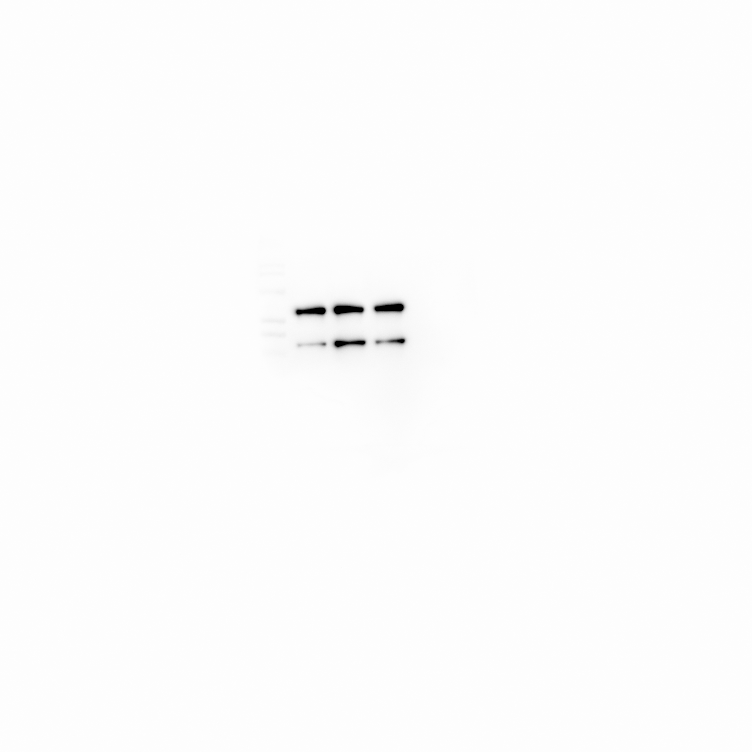


### Marker


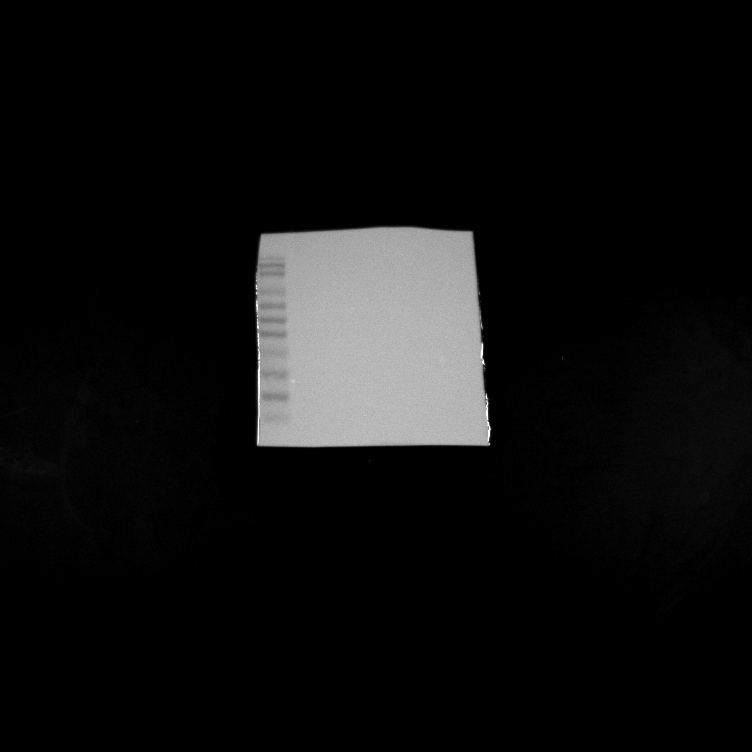

Supplement: Supplementary Table S1 — Complete list of 59 intersecting target genes between PCB2 potential targets and SCI-related genes. [file Table_1.docx]
